# Investigating the effects of complement loci CFH and ARMS2/HTRA1 on Alzheimer Disease

**DOI:** 10.1002/alz70855_106578

**Published:** 2025-12-24

**Authors:** Noel C. Moore, Yeunjoo E. Song, Alex V. Gulyayev, Kristy L. Miskimen, Renee A. Laux, Audrey Lynn, Sarada L. Fuzzell, Sherri D. Hochstetler, Dawn Miller, Laura J. Caywood, Jason E. Clouse, Sharlene D. Herington, Weihuan Wang, Ping Wang, Yining Liu, Daniel A. Dorfsman, Muneeswar Gupta Nittala, SriniVas R Sadda, Dwight Stambolian, Paula K. Ogrocki, Alan J. Lerner, Michael L Cuccaro, Jeffery M Vance, William K. Scott, Margaret Pericak‐Vance, Jonathan L Haines

**Affiliations:** ^1^ Department of Population and Quantitative Health Sciences, Case Western Reserve University School of Medicine, Cleveland, OH, USA; ^2^ John P. Hussman Institute for Human Genomics, University of Miami Miller School of Medicine, Miami, FL, USA; ^3^ Cleveland Institute for Computational Biology, Case Western Reserve University, Cleveland, OH, USA; ^4^ Doheny Eye Institute, University of California, Los Angeles, CA, USA; ^5^ Department of Ophthalmology, University of Pennsylvania, Philadelphia, PA, USA; ^6^ Department of Neurology, Case Western Reserve University School of Medicine, Cleveland, OH, USA; ^7^ Department of Neurology, University Hospitals Cleveland Medical Center, Cleveland, OH, USA; ^8^ Dr. John T. Macdonald Foundation Department of Human Genetics, University of Miami Miller School of Medicine, Miami, FL, USA

## Abstract

**Background:**

Late‐onset Alzheimer Disease (LOAD) shares multiple pathologic features and genetic risk factors with Age‐related Macular Degeneration (AMD). Amyloid‐beta (Ab) forms amyloid plaques in the brain and aggregates with other proteins and lipids to form drusen deposits in the retina of AMD eyes. *CFH* and *HTRA1*, genes coding for Ab‐processing complement proteins, are the strongest genetic risk factors for AMD, but the association with LOAD has been equivocal. In addition, the *APOE* e4 allele, LOAD's strongest genetic risk factor, has the opposite effect (e.g. is protective) for AMD. Therefore, we investigated whether the strongest genetic risk factors for AMD, *CFH* and *ARMS2/HTRA1* also influence risk of LOAD.

**Method:**

Utilizing our large dataset of mid‐Western Amish individuals, we performed single nucleotide polymorphism (SNP) association analysis on the *ARMS2/HTRA1* and *CFH* loci to determine their association with LOAD. This analysis included 152 LOAD cases and 746 cognitively unimpaired controls, all evaluated by consensus review of clinical test results. Those with known AMD were excluded from this study.

**Result:**

Our preliminary results found no significant association between LOAD and the individual SNPs defining the *CFH* or *ARMS2/HTRA1* loci. As these genes are in regions of strong linkage disequilibrium, these SNPs define a small set of extended haplotypes, which have known differential impact on AMD.

**Conclusion:**

Single SNP association analyses did not expose any significant associations between LOAD and single SNPs at the *CFH* or *ARMS2/HTRA1* loci. Examining the haplotype association with LOAD will allow for increased power and better understanding of the risk these two loci confer to LOAD.

